# The Influence of Antenatal Oral Iron and Folic Acid Side Effects on Supplementation Duration in Low-Resource Rural Kenya: A Cross-Sectional Study

**DOI:** 10.1155/2020/9621831

**Published:** 2020-05-11

**Authors:** Shadrack Oiye, Margaret Juma, Silvenus Konyole, Fatuma Adan

**Affiliations:** ^1^Intergovernmental Authority on Development (IGAD), Djibouti; ^2^University of Nairobi Institute of Tropical and Infectious Diseases (UNITID), Nairobi, Kenya; ^3^Great Lakes University of Kisumu, Kisumu, Kenya; ^4^Department of Nursing, Kenya Medical Training Centre, Nairobi, Kenya; ^5^Department of Nutritional Sciences, Masinde Muliro University of Science and Technology, Kakamega, Kenya

## Abstract

**Background:**

Undesirable effects of a daily regimen of iron and folic acid ingested jointly (iron-folate) are potential disincentives to optimal antenatal supplementation. We intended to profile antenatal iron-folate side effects and elucidate their influence on supplementation duration in low-resource rural Kenya.

**Methods:**

This was a cross-sectional descriptive study of randomly selected postnatal mothers of under-five-year-old children. Using a modified WHO Safe Motherhood Assessment standard questionnaire, they recalled the total number of days of antenatal iron-folate intake and the attendant supplement-attributed undesirable experiences. The analyses considered only participants who ingested the supplements in their immediate last pregnancies (*n* = 277).

**Results:**

About half of the study participants reported at least a side effect and a mean of 2.4 (SD 1.5) effects per person in the entire pregnancy period. Most common reported effects were chest pains (31.8%), constipation (28.5%), severe stomach pains (11.6%), and diarrhoea (11.6%). Mothers who reported at least a side effect ingested the supplements for ten days less compared to those who did not experience any effect (*p* = 0.03); and a greater proportion of the former were primigravida (*p* = 0.02) and used combined form of iron and folic acid (*p* = 0.003). In a multivariate analysis, significant correlations with supplementation compliance (ingestion for 90+ days) were found only for nausea and severe stomach pain experiences (*r* = −0.1, *p* = 0.04; *r* = 0.2, *p* = 0.01, resp.).

**Conclusions:**

The commonness of undesirable experiences attributed to daily ingestion of 60 mg iron and 0.4 mg folic acid and their deterrence to longer supplementation durations suggest the need for considering a weekly intermittent regimen for some antenatal women in such set-ups. Our study demonstrated that potentially, more counselling on nausea as a side effect might be critical in advancing iron-folate supplementation compliance.

## 1. Introduction

Antenatal iron and folic acid (iron-folate) supplementation is one of the critical public health measures for enhancing positive pregnancy outcomes by forestalling maternal anaemia, puerperal sepsis, low birth weight, and preterm birth [1]. Iron supplementation replenishes maternal stores mobilised for fetal development [2, 3] through augmentation of maternal haemoglobin mass expansion and erythrocyte quantity [3, 4]. The World Health Assembly has set a global target of reducing anaemia in women of reproductive age by 50%, by the year 2025, and antenatal iron supplementation is among the cost-effective interventions in achieving this goal (WHO 2014). Folic acid supplementation is protective against fetal neural tube defects and maternal anaemia. At the time of this study, the World Health Organization (WHO) recommendation was a daily intake of 30 to 60 mg of elemental iron and 400 *μ*g (0.4 mg) of folic acid by all pregnant adolescents and adult women, starting as early as possible and taken throughout pregnancy as one combined supplement [5]. Later in 2016, an option of weekly 120 mg of elemental iron and 2.8 mg folic acid was also recommended [6]. Nevertheless, most sub-Saharan countries, including Kenya, have hitherto adopted the policy of daily ingestion with the combined form of the supplement.

The duration of iron-folate supplementation is indicative of compliance and can predict a positive change in serum haemoglobin [7, 8]. *Ad hoc* assessments and Demographic Health Surveys (DHS) collect data on iron-folate supplementation coverage and duration, where a minimum of 90 days of antenatal ingestion is a critical cut-off [9]—a proxy indicator of compliance. In 2008, Kenya reported that only 2.5% of pregnant women ingested the supplements for 90+ days, and this increased marginally to 7.5% in 2014 [9, 10]. In the present study location, 18.3% ingested the iron-folate for 90+ days [11]. In Ethiopia, Uganda, and Tanzania (neighbouring countries to Kenya), the current figures are similarly depressed at 5.1%, 22.6%, and 21.4%, respectively [12–14].

Countries and programs are sensitive to deterrents of service uptake and compliance—whether they are innately related to the benefactors or operating health systems. In low-resources areas, iron-folate distribution and utilisation present unique bottlenecks due to the multiple interconnected issues of supplies, coverage, maternal acceptance, knowledge, attitude, and compliance, among other things. These supplements are offered free or subsidised in sub-Saharan Africa, but silent maternal costs may include the attendant undesirable experiences—the side effects.

Ministries of health and programs in sub-Saharan Africa put up efforts to enhance iron-folate supplementation coverage, acceptance, and compliance. The understanding of the burden of supplementation side effects and the programmatic implications is thus imperative. Location- and context-specific data are also needed to indicate if weekly supplementation, which elicits fewer side effects [15–17], should be considered for some antenatal women. Consistent with other medical formulations, side effects attributed to iron-folate supplements deter compliance in use. What has not extensively been studied is the relative occurrence of and how specific distresses independently influence the supplementation duration. We thus analysed the distribution of the antenatal iron-folate side effects and their influences on supplementation duration and compliance.

## 2. Subjects and Methods

### 2.1. Study Design and Setting

Data were extracted from a study which investigated the general factors influencing the duration of antenatal iron-folate supplementation [11]. This present analysis excluded from the original data set participants who did not consume iron-folate supplements during the entire antenatal period. We analysed the influence of the associated undesirable effects on the duration of supplementation. This study was a descriptive cross-sectional study among women with infants and young children aged 0-60 months attending mother-child health (postnatal) clinics. The study area, Kalama Ward (a division of Machakos district at the time of the study), is located in the present Machakos County in the lower Eastern Kenya, a low-resource rural set-up. Presently, Kenya is divided into 47 devolved semiautonomous counties, each divided into wards, which predevolution were divisions of districts.

### 2.2. Sample Size

Sample size computation was as described by Gibson and Ferguson [18] and as detailed before [11]. Briefly, key parameters in sample size computation were estimated mean and standard deviations of antennal supplementation days. A sample size of 66 per child age group (0-11, 12-23, 24-35, 36-47, and 48-60) was derived. With a contingency consideration of 5%, the total sample size was 346. Sixty-nine cases (~20%) were omitted from the analysis since they did not ingest iron-folate supplements in their last pregnancies—leaving 277 cases for analysis.

### 2.3. Sampling of the Participants

Participants were selected from all the seven health facilities in the study location as explained elsewhere [11]. In summary, the numbers drawn from each health facility were proportionate to the estimated average daily facility antenatal attendance. Participants were randomly selected from each child age group using random tables.

### 2.4. Exclusion Criteria

Underage (<18 years of age) mothers were excluded due to ethical reasons. Guardians accompanying the children to the health facilities were also not considered. Only biological mothers were interviewed.

### 2.5. Data Collection

The WHO Safe Motherhood Assessment tool [19] was adjusted and pretested for the study objectives. Each woman was asked to recall the total number of days she took iron-folate supplements in her entire immediate previous pregnancy period, a question akin to the DHS one. The number of days recalled was either continuous or intermittent, but our tool did not provide a distinction between the two types. Participants were shown tablets and syrup forms of the supplements to aid remembrance. Each participant was asked if she experienced the following side effects: nausea, vomiting, constipation, diarrhoea, black stools, stomach cramps, severe stomach pain, heartburn, chest pains, blue colour on the lips and nails, and clumsy skin. Other data sets collected included maternal sociodemographic characteristics, gestational age at the first visit to the clinic for antenatal services, number of antenatal visits, maternal knowledge on iron-folate supplementation, supplement form (tablet or syrup), and supplement combination (separate iron and folic acid or combined form).

### 2.6. Statistical Analysis

Normality of each continuous variable was tested using the Shapiro-Wilk test. The Mann-Whitney *U* test for independent variables was used to determine statistical differences for nonnormal continuous variables; otherwise, Student's *t*-test for independent variables was used. The number of days of supplementation had nonnormal distribution. Phi and Cramer's statistics was used to detect the differences in categorical variables. In the DHS analyses, 90+ days of ingestion denoted optimum supplementation, and we adopted this cut-off as an indication of compliance. The association between a reported specific side effect and 90+ days of supplementation (compliance) was analysed by correlating the two in both univariate and multivariate models and controlling for ANC visits, gestational age of first ANC visit, knowledge of supplementation scheduling, postpartum period (age of the child), and mother support group participation. These factors had previously been found to influence supplementation compliance [11]. All statistical significances were tested at *α* = 0.05.

## 3. Result

### 3.1. Participant Characteristics and Overall Supplementation Duration

About half of the antenatal mothers who ingested iron-folate supplements (*n* = 138) experienced at least a side effect. Maternal general characteristics are as shown in Table 1, disaggregated by the side effect experiences: mothers who reported at least a side effect (MRSE) versus mothers who did not report a side effect (MNRSE). The mean ages and the number of children of the two groups were comparable. Compared to MNRSE, a greater proportion of the MRSE were primigravida and the difference was statistically significant. Both groups were mostly in marital relationships and of Christian faith. Slightly less than half of them had attained secondary education and beyond. Most mothers were not employed and were not active members of mother support groups in their respective locales.

As shown in Table 1, MNRSE (compared to MRSE) took the supplements for ten more days, and the difference in the mean supplementation days was statistically significant (*p* = 0.04). An MRSE experienced, on average, 2.4 side effects. The proportion of those who ingested the supplements for 90+ days was comparable between the two groups (*p* = 0.8). Majority of the mothers used tablet forms, and a higher proportion of MRSE ingested the combined formulation of the supplement (iron and folic acid together) as compared to the MNRSE group (*p* = 0.003). The proportions of those who initiated supplementation in their first trimester, as indicated by antenatal clinic attendance in this trimester, were comparable between the two groups.

### 3.2. Distribution of Side Effects and Duration of Supplementation

Table 2 depicts the distribution of undesirable experiences attributed to ingested iron-folate. Proportions of antenatal women who experienced chest pains (31.8%) and constipation (28.5%) were disproportionately higher than those of other experiences. Experiences of severe stomach pains, diarrhoea, nausea, and heartburn were moderate (11.6%, 11.6%, 7.6%, and 7.2%, resp.). Least commonly experienced were blue colour lips and fingernails, vomiting, stool discolouration, stomach cramps, and clumsy skin. Table 2 also compares the mean days of supplementation by the antenatal distresses. Overall, MNRSE ingested the supplements for a relatively longer duration compared to MRSE. However, the differences in lengths of supplementation were statistically significant only for constipation, diarrhoea, black stool, and heartburn (*p* < 0.05).

### 3.3. Correlation of Side Effects with Supplementation Compliance

Table 3 shows the correlation between specific side effects experienced and supplementation compliance—intake for a minimum of 90 days, antepartum. In univariate analysis, only severe stomach and chest pains had a positive and significant correlation with compliance. When controlled for the number of ANC visits, gestational age of first ANC visit, knowledge of supplementation scheduling, postpartum period (age of the child), and mother support group involvement, a significant correlation was found for nausea (*r* = −0.1, *p* = 0.04) and severe stomach pains (*r* = 0.2, *p* = 0.01).

## 4. Discussion

We demonstrated the sensitivity of duration of antenatal iron-folate supplementation to a host of attendant individual distresses. We also showed that concurrent daily ingestion of 60 mg of elemental iron and 0.4 mg folic acid provoked the experience of about ~2 side effects per antenatal mother, on average. Several specific distresses prompted significant declines in the days of antenatal supplementation, and only nausea had a significant negative correlation with compliance.

Nausea, constipation, diarrhoea, heartburn, and vomiting are common side effects of supplemental iron [20, 21]. At the same time, high doses of folic acid may cause abdominal cramps, diarrhoea, rash, sleep disorders, irritability, confusion, nausea, stomach upset, behaviour changes, skin reactions, seizures, gas, and excitability, among others [22]. In combination, iron and folic acid supplements elicit experiences of nausea, diarrhoea, heartburn, constipation, and vomiting [21]. It is thus apparent that specific side effects reported in this study were not peculiar and were confirmed by an online medical source [23]. At the time of this study, Kenya was in a policy transition, from single to combined form of the supplement. Some antenatal women, therefore, were still on separate supplements and more likely to miss one or other. These women were expected to experience fewer side effects compared to counterparts who were on combined forms. This may explain why in this present study, a higher proportion of those who did not experience side effects took single types of the supplement compared to those who reported the discomforts.

To our knowledge, this study was probably one of the first in sub-Saharan Africa to estimate the mean number of side effects experienced per antenatal woman. We also showed that a first-time mother was more likely to report the experiences as compared to a multigravida. The newness of the supplement to a primigravida may explain their higher likelihood to experience the distresses. Perhaps, first-time mothers also had lesser exposure to antenatal counselling *vis-a-vis* the veterans. The intake interval is also a factor in experiencing supplementation upsets [6], and all the study participants were on the daily regimen. Weekly supplementation for antenatal women [6] can minimise the side effects experienced, but this intake interval is not an option in Kenya.

Antenatal experience of at least a side effect resulted in significantly lesser days of supplementation compared to those who did not report any side effect. In an earlier analysis [11], however, this variation was not observed mainly due to the inclusion in the analysis participants who did not ingest the supplements at all. Not all side effects elicited a significant decline in days of supplementation. Nevertheless, we could not decipher why out of the 11 side effects reported, only constipation, diarrhoea, black stool, and heartburn were associated with lesser days of supplementation. We could only postulate that potentially these were least tolerated by antenatal mothers.

Findings of limited iron-folate supplementation compliance due to associated side effects have been reported in different set-ups [25–27]. A previous Kenyan study had also indicated that generally, the distressful experiences did not significantly influence compliance, but their management was favourable over seven days of antenatal follow-ups [28]. Further, access to antenatal services and the close follow-ups which integrate counselling on management of side effects have been shown pertinent in improving compliance [11, 24]. Our current analysis presents a different perspective in that we analysed for compliance, not by general experience, but by specific side effects during the entire antenatal period. The depiction of significant negative correlation between nausea and compliance was comparable to that of Bangladeshi [29] and South African antenatal women [30]. Nausea may have other causes in pregnancy, but irrespective, the distress may reduce dietary [31] and other oral intakes including supplements—and especially if the supplements themselves are perceived to be the source of the distresses.

This study had some limitations. It was not possible to isolate if a side effect was due to iron-folate or ingestion of other medications or food intakes or even due to hormonal pregnancy changes. Possible recall errors did not elude the study as we adopted the DHS question of recalling days of supplementation in cross-sectional studies. The question was phrased: During the whole pregnancy, for how many days did you take the iron-folate tablets or syrup supplement? To minimise potential mix-up with other medications by participants, they were shown common forms of iron and folic acid supplements dispensed in the study area.

## 5. Conclusion

Side effects of daily concurrent ingestion of 60 mg of elemental iron and 0.4 mg folic acid account for a loss of about ~10 days of antenatal supplementation. Since half of the women experienced the side effects, and hitherto daily ingestion results in relatively more side effects, the weekly intermittent regimen might be a critical consideration for some antenatal women in such set-ups. Nausea was the only distress negatively correlated with the supplementation compliance, albeit possible confounds by other nausea triggers during pregnancy. Awareness on the expected experience of nausea and antepartum counselling on its management might be critical in reducing cases of iron and folic acid supplementation noncompliance.

## Figures and Tables

**Table 1:** Maternal characteristics and supplementation profile.

General characteristics	Proportions/values			
	All (*n* = 277)	^∞^MRSE (*n* = 138)	^∞^MNRSE (*n* = 139)	*p* value^∗^
Mean age in years ± SD	28.2 ± 5.2	27.9 ± 5.1	28.5 ± 5.2	0.26
Mean number of children ± SD	1.7 ± 0.5	1.6 ± 0.5	1.8 ± 0.4	0.02
Proportion primigravida (% (*n*))	31.8 (88)	40.6 (56)	23.0 (32)	0.002
Proportion married (% (*n*))	79.5 (220)	78.3 (108)	80.7 (109)	0.61
Proportion Christians (% (*n*))	98.2 (272)	99.1 (105)	97.3 (108)	0.33
Proportion with secondary education and above (% (*n*))	44.9 (124)	46.0 (63)	43.8 (60)	0.71
Proportion in salaried employment (% (*n*))	9.0 (25)	9.6 (13)	9.2 (12)	0.30
Proportion belonging to mother support group (% (*n*))	13.7 (38)	13.8 (19)	13.7 (19)	0.98
Proportion of mothers who attended antennal clinic for the first time in the first trimester (% (*n*))^¥^	47.3 (131)	51.4 (71)	43.2 (60)	0.17
Mean days of supplementation ± SD	48.1 ± 35.7	43.3 ± 30.7	52.9 ± 40.0	0.04
Mean days of supplementation by the postpartum period of recall ± SD^*α*^				
0-11 mo	48.0 ± 38.0	43.7 ± 31.7	51.3 ± 42.6	0.09
12-23 mo	57.7 ± 30.2	59.4 ± 30.4	55.5 ± 30.7	
24-35 mo	53.0 ± 41.5	50.7 ± 33.0	55.8 ± 50.5	
36-47 mo	40.3 ± 28.1	31.7 ± 25.8	46.4 ± 28.4	
48-60 mo	44.0 ± 37.0	31.6 ± 24.0	58.9 ± 44.1	
Mean number of side effects experienced ± SD	1.2 ± 1.6	2.4 ± 1.5	—	—
Proportion optimally supplemented (for 90+ days)—compliance (% (*n*))	23.1 (64)	23.9 (33)	22.3 (31)	0.75
Tablet (not syrup) form of iron-folate ingested (% (*n*))	94.9 (263)	94.2 (130)	95.7 (133)	0.57
Combined formulation (% (*n*))*^Ω^*	44.8 (124)	58.6 (74)	36.0 (50)	0.003

*^∞^MRSE = mothers who reported at least a side effect; MNRSE = mothers who did not report a side effect.* ^∗^ *p* value for statistical comparison of MRSE versus MNRSE. For continuous data with normal distribution, Student's *t*-test for independent samples was used. For continuous data with nonnormal distribution, Mann-Whitney *U* test for independent variables was used. For mean days of supplementation by the recall for different age groups, *p* values are based on Analysis of Variance (ANOVA) used. For all categorical variables, Phi and Cramer's *V* statistics (*α* = 0.05) was used. ^¥^Attendance of ANC in the first trimester is an indication of receiving the iron and folic acid supplements earlier, than later in pregnancy. ^*α*^As indicated by the age of their last-born child, and this reflects the time since the end of the last pregnancy in months. *^Ω^*Iron and folic acid in the same tablet of syrup formulation. SD = standard deviation (*α* = 0.05); mo = months.

**Table 2:** Distribution of side effects and number of days of supplementation by effect.

Specific side effects	% of mothers reporting side effects (*n* = 277)	Mean number of days of supplementation		*p* value^∗^
		^∞^MRSE (*n* = 138)	^∞^MNRSE (*n* = 139)	
Nausea	7.6	35.8 ± 31.8	49.0 ± 35.9	0.06
Vomiting	5.1	32.7 ± 26.2	49.0 ± 35.9	0.07
Constipation	28.5	39.0 ± 29.9	51.7 ± 37.2	0.002
Diarrhoea	11.6	37.2 ± 32.0	49.5 ± 36.0	0.01
Black stool	4.7	26.4 ± 24.7	49.1 ± 35.8	0.01
Stomach cramps	2.5	26.0 ± 21.2	48.6 ± 35.8	0.09
Severe stomach pains	11.6	57.4 ± 34.7	46.7 ± 35.7	0.06
Heartburn	7.2	29.6 ± 22.2	49.5 ± 36.1	0.01
Chest pains	31.8	46.7 ± 33.5	48.7 ± 36.8	0.5
Blue colour lips and fingernails	5.4	44.0 ± 40.1	48.3 ± 35.5	0.4
Clumsy skin	2.5	43.3 ± 28.6	48.3 ± 35.9	0.9

^∗^Based on the Mann-Whitney *U* test for independent variables. *^∞^*MRSE = mothers who reported at least a side effect; MNRSE = mothers who did not report a side effect.

**Table 3:** Correlation between reported side effects and iron-folate compliance (supplementation for 90+ days).

Side effects	Coefficient of correlation (*r*) (*n* = 277)			
	Univariate analysis	*p* value	Multivariate analysis^∞^	*p* value
Nausea	-0.06	0.32	-0.1	0.04
Vomiting	-0.05	0.42	-0.1	0.08
Constipation	-0.04	0.48	-0.06	0.34
Diarrhoea	-0.01	0.86	-0.02	0.80
Black stool	-0.08	0.18	-0.1	0.13
Stomach cramps	-0.09	0.14	-0.1	0.24
Severe stomach pains	0.2	0.001	0.2	0.01
Heartburn	-0.1	0.05	-0.1	0.08
Chest pains	0.1	0.04	0.03	0.54
Blue colour lips and fingernails	0.0 6	0.34	-0.01	0.86
Clumsy skin	-0.03	0.57	-0.07	0.29

^∞^Controlling for the number of ANC visits, gestational age of first ANC visit, knowledge of supplementation scheduling, postpartum period (age of the child), and mother support group participation.

## Data Availability

The datasets for this study are available from the corresponding author on reasonable request.

## Abbreviations

DHS: Demographic Health Surveys
MNRSE: Mothers who did not report a side effect
mo: Months
MRSE: Mothers who reported at least a side effect
NACOSTI: National Commission of Science, Technology and Innovation
*p*: *p* value
*r*: Coefficient of correlation
SD: Standard deviation
SPSS: Statistical Package for Social Sciences
WHO: World Health Organization.
